# Effect of commercial fibrolytic enzymes application to normal- and slightly lower energy diets on lactational performance, digestibility and plasma nutrients in high-producing dairy cows

**DOI:** 10.3389/fvets.2024.1302034

**Published:** 2024-05-03

**Authors:** Jiahua Yang, Shengguo Zhao, Bo Lin

**Affiliations:** ^1^College of Animal Science and Technology, Guangxi University, Nanning, China; ^2^Key Laboratory of Quality & Safety Control for Milk and Dairy Products of Ministry of Agriculture and Rural Affairs, Institute of Animal Sciences, Chinese Academy of Agricultural Sciences, Beijing, China

**Keywords:** dairy cow, feed efficiency, fibrolytic enzyme, milk production and composition, plasma nutrients

## Abstract

The inclusion of fibrolytic enzymes in the diet is believed to have positive effects on animal production. Hence, the objective of this study was to investigate the impact of supplementing diets with a commercial fibrolytic enzyme preparation (**Vistamax**; mixture of xylanase and cellulase) derived from *Trichoderma reesei* on lactational performance, digestibility, and plasma nutrient levels in high-producing dairy cows. Two dietary energy levels were considered: a normal energy diet (metabolizable energy = 2.68 Mcal/kg) and a slightly lower energy diet (metabolizable energy = 2.55 Mcal/kg). A total of 120 lactating Holstein cows (parity = 2; Days in Milk = 113 ± 23) were randomly assigned to four treatment groups using a 2 * 2 factorial arrangement. The dietary treatments consisted of: (1) normal energy diet without enzyme supplementation (NL); (2) normal energy diet with enzyme supplementation (NLE); (3) slightly lower energy diet without enzyme supplementation (SL); and (4) slightly lower energy diet with enzyme supplementation (SLE). The amount of enzyme added to the diets was determined based on previous *in vitro* studies and supplier recommendations. The enzyme and premix were mixed prior to the preparation of the total mixed ration, and the trial lasted for a duration of 42 days. The results indicated that the application of the fibrolytic enzyme did not have a significant effect on dry matter intake (DMI), but it did enhance the digestibility of dry matter (DM), neutral detergent fiber (NDF), potentially digestible NDF (pdNDF), organic matter (OM), milk production, milk urea nitrogen (MUN), and blood urea nitrogen (BUN). On the other hand, the slightly lower energy diet resulted in a decrease in DMI, milk production, milk protein yield, plasma free amino acids (FAA), and an increase in plasma B-hydroxybutyrate (BHBA). In conclusion, the inclusion of the fibrolytic enzyme in the diets of dairy cows led to improvements in the digestibility of DM, NDF, pdNDF, OM, milk production, and feed efficiency. Furthermore, the application of the enzyme to the slightly lower energy diet resulted in milk production levels comparable to those observed in cows fed the untreated normal energy diet.

## 1 Introduction

Corn grain prices have exhibited an upward trend in recent years, and projections suggest that this trend will continue in the future (1). Consequently, the formulation of reduced-starch diets has become economically advantageous and serves to mitigate the risk of rumen acidosis (2). One common approach to reduce dietary starch is the partial substitution of grain with highly digestible forage (3). However, the rate and extent of forage digestion in dairy cows are generally lower compared to concentrates, limiting feed intake and overall performance. As the reduction of starch content in the diet occurs, the dietary energy may become insufficient to sustain the high production performance of ruminants. Consequently, increasing the digestibility of dietary fiber has emerged as a strategy to provide additional energy for ruminants. Therefore, the inclusion of fibrolytic enzymes in ruminant diets to enhance forage digestibility becomes crucial for increasing milk production.

Fibrolytic enzymes play a significant role in improving the digestibility of forage fiber and enhancing the lactation performance of dairy cows. A meta-analysis revealed that fibrolytic enzymes typically enhance the digestibility of neutral detergent fiber (NDF) and dry matter (DM) throughout the digestive tract, resulting in a relatively modest increase in milk production (4). Several studies have reported the positive impact of fibrolytic enzymes on the digestibility of DM and NDF in ruminant diets (5–7). Moreover, numerous investigations have demonstrated that the addition of fibrolytic enzymes to ruminant diets can improve the lactational performance of dairy cows (8–11). However, there have been inconsistent findings regarding the effect of cellulase on dairy cow performance (12–14). These discrepancies can be attributed to various factors, such as variations in enzyme activity, application rate, method of application, substrate specificity, and stage of lactation in dairy cows (15, 16). Another factor influencing the divergence in study outcomes is the basal diet to which the enzymes are added. A diet with high energy content comprising highly digestible feed ingredients, such as starch, may derive less benefit from the inclusion of exogenous enzymes (16). Thus, a low energy diet with a higher proportion of forage may provide a clearer understanding of the effects of fibrolytic enzymes compared to a highly digestible diet. However, fewer studies have been conducted on the effects of adding fibrolytic enzymes to normal and slightly lower energy diets on the performance of high yielding dairy cows. Therefore, the objectives of this study were to evaluate the effects of incorporating fibrolytic enzymes into normal and slightly lower energy diets for high-yielding dairy cows and to develop strategies for reducing starch intake in dairy cow feeding practices in China.

## 2 Materials and methods

### 2.1 Enzyme product

The enzyme preparations utilized in this study were obtained from *Trichoderma reesei* (**Vistamax**; AB Vista, Wiltshire, United Kingdom). The product consists of a combination of xylanase (EC 3.2.1.8; xylanase activity ≥17,325 BXU/g, where one BXU represents the amount of enzyme capable of releasing 0.06 micromole of xylose equivalent from birchwood xylan per minute) and cellulase (EC 3.2.1.8; endoglucanase activity ≥8,250 ECU/g, where one ECU represents the amount of enzyme that can release 0.06 micromole of glucose from medium-viscosity carboxymethylcellulose per minute). Prior to the commencement of the experiment, the enzymes were thoroughly mixed with the premix at a rate of 1,000 g/t DM feed based on dry matter intake (DMI), following the instructions provided on the product label. The premixes containing the enzymes were directly applied to the total mixed ration (TMR) in mixer trucks.

### 2.2 Cows, treatments, and design

A total of 120 mid-lactation Holstein cows (parity = 2; DIM = 113 ± 23) were randomly assigned to one of four treatment groups, arranged in a 2 * 2 factorial design. The cows were fed either a normal energy diet or a slightly lower energy diet, with or without the addition of fibrolytic enzymes. The following treatments were investigated: (1) normal energy untreated diet (NL, metabolizable energy = 2.68 Mcal/kg); (2) normal energy diet with enzyme supplementation (NLE); (3) slightly lower energy untreated diet (SL, metabolizable energy = 2.55 Mcal/kg); (4) slightly lower energy diet with enzyme supplementation (SLE). The cows were provided *ad libitum* access to the TMR, with a 5% allowance for refusal. The diets were formulated using NDS Professional ration formulation software (version 3, RUM&N, NDS Professional, Reggio Nell’Emilia, Emilia-Romagna, Italy) to ensure an adequate supply of metabolizable protein and either adequate or slightly lower metabolizable energy for mid-lactation dairy cows, following the guidelines of the National Research Council (2001). The normal energy diet consisted of 32.05% corn silage, 9.37% alfalfa hay, 5.52% distillers dried grains with soluble (DDGS), and 53.06% concentrate (dry matter basis). The slightly lower energy diet consisted of 33.05% corn silage, 9.87% alfalfa hay, 6.02% DDGS, and 51.06% concentrate (DM basis). The detailed composition of the TMR is presented in Table 1. The experiment was conducted from November 12, 2022, to January 7, 2023, at Yuran Dairy Farm, Pingdingshan City, Henan Province, China, with a duration of 42 days. The cows were allowed a 14-day adaptation period to the diets, and the last 28 days of the trial were designated for sample collection. The cows were housed in semi-enclosed barns with windows, which were closed when the ambient temperature fell below 5°C to prevent cold stress. Fresh clean water and mineral salt were continuously available, and an automatic water heater was used in the drinking trough to maintain the water temperature at 16°C. An adequate number of freestalls, at least one per cow, were provided with loose bedding and underwent daily maintenance.

**Table 1:** Diet composition and nutrient composition of diets.

	Normal energy		Slightly lower energy	
Items	Control	Enzyme	Control	Enzyme
Ingredient, g/kg DM				
Corn silage	32.05	32.05	33.05	33.05
Alfalfa hay	9.37	9.37	9.87	9.87
Ground corn	12.54	12.54	12.54	12.54
Flaked corn	12.19	12.19	10.19	10.19
Soybean meal	8.41	8.41	8.41	8.41
Extruded soybean	1.08	1.08	1.08	1.08
Soy hulls	1.44	1.44	1.44	1.44
Canola meal	3.52	3.52	3.52	3.52
Whole cotton seed	6.12	6.12	6.12	6.12
DDGS	5.52	5.52	6.02	6.02
Fat powder	0.47	0.47	0.47	0.47
Sodium bicarbonate	0.60	0.60	0.60	0.60
Molasses	2.40	2.40	2.40	2.40
Premix^1,2^	4.29	4.19	4.29	4.19
ABV	-	0.10	-	0.10
Particle size distribution, % as fed				
>19 mm	1.1	0.8	0.6	1.2
19–8 mm	41.5	39.6	40.3	41.6
8–4 mm	17.5	18.1	16.4	17.1
<4 mm	39.9	41.6	42.7	40.0
Chemical composition, % of DM				
DM, %	51.60	51.60	50.67	50.67
ME, Mcal/kg	2.68	2.68	2.55	2.55
CP,%	16.92	16.92	17.04	17.04
peNDF,%	19.06	19.06	19.47	19.47
NDF,%	26.83	26.83	27.39	27.39
Starch,%	28.70	28.70	27.59	27.59
Ether extract,%	6.42	6.42	6.46	6.46

^1^Content per kilogram of product: 78.8 g of Ca, 20 mg of Co, 280 mg of Cu, 26 mg of I, 36.2 g of Mg, 1,100 mg of Mn, 10 mg of Se, 79 g of Na, 1,500 mg of Zn, 100,000 IU of vitamin A, 30,000 IU of vitamin D3, 1,600 IU of vitamin E. ^2^Composition: Yeast Culture XP = 2%; Optigen (Non-protein nitrogen) = 8%; RPLys = 2.5%; RPMe = 1.5%.

### 2.3 Sampling and analysis

The cows were fed two equal meals per day, with feedings occurring at 07:00 and 14:00 h. The daily dry matter intake (DMI) for each herd was recorded, taking into account the weight of feed delivered by the mixer wagon minus the weight of leftovers collected by the leftover cleanup trucks. The DMI per cow was determined by dividing the DMI per shed by the number of cows in the pen on that particular day. Twice a week, samples of feed and residual feed from each treatment were collected, mixed together, and stored at −20°C for further analysis.

The cows were milked three times a day, with milking sessions at 06:30, 12:30, and 18:30 h. Daily milk production was recorded using milking equipment from Delaval, Sweden. Milk samples were collected at the 21st and 42nd day of the trial, with the morning, afternoon, and evening milk volumes collected in a ratio of 4:3:3. The milk samples were stored at 4°C with the addition of bronopol-B2 preservative from D&F Control System Inc., Dublin, ON, Canada. The mean of the two milk composition assessments was employed as the representative milk composition for the duration of the testing period. Milk fat, protein, and urea nitrogen were analyzed using a mid-infrared machine (Foss MilkoScan, Foss Food Technology Corp., Eden Prairie, MN, United States). Milk somatic cell count (SCC) was measured using an SCC analyzer (Fossomatic 7/7 DC, FOSS). Fat-corrected milk (FCM), energy-corrected milk (ECM), and the yields of milk protein, lactose, and fat were calculated following the method described by Refat (10).

Blood was collected via the tail vein in 10 mL sodium heparin anticoagulant tubes (Becton Dickinson, Rutherford, NJ) 1 h after morning feeding at the test site on 42d. Plasma was subsequently stored at −80°C for subsequent testing. Glutamicacid (GLU), triglyceride (TG), high-density lipid (HDL), low-density lipid (LDL), total cholesterol (TC), uric acid (UA), creatinine (CR), free amino acid (FAA), blood urea nitrogen (BUN), and β-hydroxybutyric acid (BHBA) levels in plasma were analyzed using an automatic plasma analyzer (Mindray BS200, Shenzhen, China).

Fecal samples were collected from the rectum of 30 cows in each group in the morning before feeding, and 10 mL 10% sulfuric acid (v/v) was added immediately into per 100 g fecal sample to mix together. The fecal samples weighing between 300 and 500 g on a fresh basis were collected every 6 h for 3 days on day 40, day 41, and day 42, then fecal samples from six cows were mixed within each group. All frozen TMR, refusals, and fecal samples were thawed overnight at room temperature and then dried at 55°C for 48 h. The dried samples were ground to 1 mm using a Wiley mill (A. H. Thomas, Philadelphia, PA) and sent to the Chinese Academy of Agricultural Sciences (CASA, Beijing, China) for chemical analysis, including measurements of dry matter (DM) using AOAC(2000) method 930.15, ash using AOAC(2000) method 942.05, crude protein (CP) using AOAC(2000) method 990.03, neutral detergent fiber (NDF) using the method described by Van Soest (17), and starch using the method described by Hall (2009). The NDF contents of the feed and orts were determined using heat-stable amylase (type XI-A of *Bacillus subtilis*; Sigma-Aldrich, St. Louis, MO, United States), and anhydrous sodium sulfate was added during the boiling progress, the final NDF content was incorporated ash in this study. The equation of energy value of feeds was calculated as follows:

$DE\left(Mcal/kg\right)=0.04409\times TDN\left(\%\right)$ ;

$ME\left(Mcal/kg\right)=0.82DE\left(Mcal/kg\right)$ .

DE, Digestible Energy; ME, Metabolizable Energy.

Total-tract pdNDF digestibility was calculated according to Fustini (18). The digestibility of pdNDF was expressed as follows:


$$\begin{array}{l} \text{pdNDF digestibility},\%\text{pdNDF}=\\ 100-[(\text{dietary iNDF}/\text{fecal iNDF})\times \\ (\text{fecal pdNDF concentration}/\text{dietary pdNDF})]. \end{array}$$

peNDF=NDF−iNDF

pdNDF, Potentially digestible NDF; iNDF, indigestible NDF; peNDF, Physically Effective NDF.

### 2.4 Statistical analysis

To assess the effect of firbrolytic enzyme and diet energy level on lactating dairy cows, data of all parameters in this study were analyzed using the PROC MIXED procedure of SAS (ver. 9.4, SAS Institute Inc.). The model encompassed fixed effects for energy level and enzyme supplementation, along with their interaction. To address the non-normal distribution of SCC data, a natural logarithm transformation was applied. In the model, LSMEANS statements were used, adjusted for multiple comparisons by ADJUST = TUKEY, to calculate least squares means for the interaction between different energy levels and enzyme supplementation levels. Treatment significance was declared at *p* ≤ 0.05, and tendencies were declared at 0.1 > *p* > 0.05.

## 3 Results

### 3.1 Feed intake and digestibility

Table 2 presents the impact of fibrolytic enzyme application and energy level on nutrient intake and digestibility. The application of fibrolytic enzyme did not have a significant effect (*p* > 0.10) on the intake of dry matter (DM), neutral detergent fiber (NDF), starch, and organic matter (OM). However, it significantly improved (*p* < 0.001) the digestibility of DM (67.98% vs. 64.96%), NDF (36.13% vs. 31.3%), potentially digestible NDF (pdNDF; 51.09% vs. 44.81%), and OM (69.98% vs. 67.96%) in the diet of dairy cows.

**Table 2:** Effect of adding a exogenous fibrolytic enzyme^1^ to diets containing normal energy (metabolizable energy = 2.68 Mcal/kg) or slightly lower energy (metabolizable energy = 2.55 Mcal/kg) on digestibility in dairy cows.

Items	Normal energy		Slightly lower energy		SEM	*p* value		
	Control	Enzyme	Control	Enzyme		Energy	Enzyme	Interaction
Intake (kg/d)								
DM	27.95^ab^	28.10^a^	27.68^b^	27.60^b^	0.075	0.010	0.818	0.457
NDF	7.50	7.54	7.58	7.56	0.020	0.206	0.824	0.460
peNDF	5.33	5.36	5.39	5.37	0.014	0.167	0.824	0.460
ST	8.02^a^	8.06^a^	7.64^b^	7.61^b^	0.028	<0.001	0.805	0.453
OM	26.00^ab^	26.13^a^	25.72^b^	25.65^b^	0.070	0.006	0.817	0.457
Digestibility (%)								
DM	65.72^b^	68.30^a^	64.20^c^	67.66^ab^	0.451	0.070	<0.001	0.442
NDF	31.96^b^	36.10^a^	30.64^b^	36.16^a^	0.671	0.438	<0.001	0.396
pdNDF	45.76^b^	51.00^a^	43.86^b^	51.18^a^	0.901	0.458	<0.001	0.371
ST	99.14	99.38	99.20	99.36	0.123	0.940	0.457	0.881
OM	68.38^b^	70.40^a^	67.54^b^	69.56^ab^	0.321	0.071	<0.001	1.000

OM, Organic matter; ST, Starch; peNDF, physically effective NDF; pdNDF, Potentially digestible NDF; DMI, DM intake. pdNDF digestibility (%) = 100 − [(dietary indigestible NDF/fecal indigestible NDF) × (fecal pdNDF concentration/dietary pdNDF)]. Different superscript letters in the same row indicate significant differences among groups (*p* < 0.05). ^1^Derived from Trichoderma reesei (**Vistamax**; mixture of xylanase and cellulase).

Slightly lower energy diets resulted in decreased intake of DM (27.64 kg/d vs. 28.03 kg/d; *p* = 0.027) and starch (7.63 kg/d vs. 8.04 kg/d; *p* < 0.001) in cows. The slightly lower energy diet also showed a tendency to reduce total digestibility of DM in the digestive tract (65.93% vs. 67.01%; *p* = 0.07) and OM (68.55% vs. 69.39%; *p* = 0.07).

### 3.2 Milk yield and composition

Table 3 illustrates the effect of fibrolytic enzyme application and energy level on milk yield and composition. The application of fibrolytic enzyme increased milk yield (42.60 kg/d vs. 41.62 kg/d; *p* = 0.036), fat-corrected milk (FCM; 44.96 kg/d vs. 43.30 kg/d; *p* = 0.036), energy-corrected milk (ECM; 48.34 kg/d vs. 46.76 kg/d; *p* = 0.034), and feed efficiency. It also reduced milk urea nitrogen (MUN) levels (15.91 mg/dL vs. 17.32 mg/dL; *p* < 0.001), and enhanced FCM per unit of dry matter intake (FCM/DMI; 1.61 vs. 1.56; *p* = 0.043), and ECM per unit of dry matter intake (ECM/DMI; 1.74 vs. 1.68; *p* = 0.038).

**Table 3:** Effect of adding a exogenous fibrolytic enzyme^1^ to diets containing normal energy (metabolizable energy = 2.68 Mcal/kg) or slightly lower energy (metabolizable energy = 2.55 Mcal/kg) on milk production and composition in dairy cows.

Item	Normal energy	Slightly lower energy			SEM	*p* value		
	Control	Enzyme	Control	Enzyme		Energy	Enzyme	Interaction
Yield (kg/d)								
Milk	42.15^ab^	43.20^a^	41.08^b^	41.99^ab^	0.24	0.016	0.036	0.884
FCM	44.05^ab^	45.94^a^	42.56^b^	43.97^ab^	0.40	0.028	0.036	0.758
ECM	47.58^ab^	49.30^a^	45.94^b^	47.37^ab^	0.38	0.016	0.034	0.850
Fat	1.81^ab^	1.91^a^	1.74^b^	1.81^ab^	0.026	0.099	0.100	0.787
Protein	1.51^ab^	1.52^a^	1.45^b^	1.48^ab^	0.10	0.023	0.230	0.656
Milk composition								
fat (%)	4.31	4.44	4.26	4.34	0.066	0.559	0.434	0.847
protein (%)	3.57	3.52	3.53	3.54	0.019	0.778	0.545	0.511
Ln SCC	1.66^b^	1.08^a^	2.13^bc^	1.68^c^	0.099	0.005	0.006	0.742
MUN (mg/dL)	17.81^a^	15.41^c^	16.82^b^	16.41^b^	0.17	0.994	<0.001	0.002
Efficiency								
FCM/DMI	1.57^ab^	1.63^a^	1.54^b^	1.59^ab^	0.014	0.157	0.043	0.948
ECM/DMI	1.70^ab^	1.75^a^	1.66^b^	1.72^ab^	0.013	0.127	0.038	0.939

FCM, Fat-corrected milk; ECM, Energy-corrected milk, Ln SCC, the natural logarithm of Somatic Cell Count.; MUN, Milk Urea Nitrogen; DMI, DM intake. Different superscript letters in the same row indicate significant differences among groups (*p* < 0.05). ^1^Derived from *Trichoderma reesei* (**Vistamax**; mixture of xylanase and cellulase).

Slightly lower energy diets decreased milk yield (41.54 kg/d vs. 42.68 kg/d; *p* = 0.016), FCM (43.27 kg/d vs. 45.00 kg/d; *p* = 0.028), ECM (46.66 kg/d vs. 48.44 kg/d; *p* = 0.016), and milk protein yield (1.47 kg/d vs. 1.52 kg/d; *p =* 0.023).

### 3.3 Plasma nutrients

Table 4 presents the effect of fibrolytic enzyme application and energy level on changes in plasma nutrients. Fibrolytic enzyme application and energy levels did not have a significant effect on total cholesterol (TC), high-density lipoprotein (HDL), low-density lipoprotein (LDL), uric acid (UA), and creatinine (CR). However, the addition of fibrolytic enzyme significantly reduced blood urea nitrogen (BUN) levels (5.57 mM vs. 6.40 mM; *p* < 0.001).

**Table 4:** Effect of adding a exogenous fibrolytic enzyme^1^ to diets containing normal energy (metabolizable energy = 2.68 Mcal/kg) or slightly lower energy (metabolizable energy = 2.55 Mcal/kg) on milk plasma nutrients in dairy cows.

Item	Normal energy		Slightly lower energy		SEM	*p* value		
	Control	Enzyme	Control	Enzyme		Energy	Enzyme	Interaction
GLU (mM)	4.08	4.09	3.99	4.02	0.023	0.099	0.723	0.930
TC (mM)	6.70	6.94	7.01	7.16	0.108	0.226	0.353	0.850
TG (mM)	0.18	0.20	0.18	0.18	0.003	0.545	0.075	0.112
HDL (mM)	3.57	3.72	3.79	3.86	0.070	0.206	0.408	0.779
LDL (mM)	4.38	4.55	4.56	4.72	0.070	0.219	0.259	0.960
UA (uM)	39.69	40.27	36.23	38.22	0.964	0.157	0.507	0.716
BUN (mM)	6.18a	5.54b	6.61a	5.59b	0.077	0.076	<0.001	0.149
CR (uM)	74.46	72.98	72.26	73.75	0.414	0.391	0.996	0.074
FFA (mM)	0.17	0.17	0.16	0.17	0.002	0.368	0.355	0.289
FAA (uM)	9.30ab	10.97a	7.89b	7.04b	0.411	0.001	0.602	0.111
BHBA (mM)	0.12ab	0.07c	0.11b	0.14a	0.004	<0.001	0.327	<0.001

GLU, Glutamicacid; TC, Serum total cholesterol, HDL, High density lipid; LDL, Low density lipoprotein; UA, Uric acid; BUN, Blood urea nitrogen; CR, Creatinine; FFA, Free fatty acid; FAA, Free amino acid; BHBA, β-hydroxybutyric acid. Different superscript letters in the same row indicate significant differences among groups (*p* < 0.05). ^1^Derived from *Trichoderma reesei* (**Vistamax**; mixture of xylanase and cellulase).

Slightly lower energy diets significantly decreased free amino acids (FAA) levels (7.47 Um vs. 10.14 Um; *p* < 0.001). Plasma beta-hydroxybutyrate (BHBA) concentrations varied with substrate energy levels following the addition of fibrolytic enzyme to the diets of dairy cows (enzyme * energy interaction, *p* < 0.001), with the normal energy group having significantly lower plasma BHBA concentrations than the slightly lower energy group (0.1 mM vs. 0.13 mM; *p* < 0.001).

## 4 Discussion

### 4.1 Feed intake and digestibility

The application of fibrolytic enzymes did not affect the intake of dry matter (DM), neutral detergent fiber (NDF), starch, and organic matter (OM) in the diets of dairy cows. However, it significantly improved the digestibility of DM, NDF, potentially digestible NDF (pdNDF), and OM. These findings are consistent with previous studies that observed similar effects of exogenous fibrolytic enzyme supplementation on NDF digestibility in dairy cows fed alfalfa-corn silage-based total mixed rations (6, 9). However, it is important to note that variations in lactation stage, feed types, and enzyme activity across different studies have led to contrasting outcomes regarding the impact of enzyme-supplemented diets on nutrient intake and digestibility. Some studies have reported positive effects of fibrolytic enzymes on dry matter intake (DMI) (8, 19), while others have reported negative effects (20, 21). For instance, Refat (10) found that the addition of fibrolytic enzyme to barley silage-based diets of dairy cows did not affect DM, OM, or NDF intake, and the response of DM, OM, and NDF digestibility to increasing fibrolytic enzyme concentration followed a cubic pattern. Similarly, Yang (14) reported that the addition of fibrolytic enzyme to whole-plant faba bean silage diets for dairy cows did not affect DM and OM digestibility, and higher enzyme concentrations actually reduced NDF digestibility (48.25% vs. 44.78%). One possible explanation for this phenomenon is that higher fiber digestibility of the feed and higher enzyme concentrations may compete with ruminal microbes for binding sites on feed particles (22).

The current trial utilized corn silage-based diets for dairy cows, which are characterized by low neutral detergent fiber (NDF) content, high starch content, and smaller particle size. These attributes contribute to faster degradation and clearance of the feed from the rumen, resulting in lower NDF degradation rates (23). In a study (24) was reported that an average of 32% of rumen NDF was degraded in corn silage, with an additional 7% being digested in the post-ruminal tract. In the present trial, the addition of fibrolytic enzyme to whole-plant corn silage-based diets for dairy cows significantly improved fiber digestibility. This improvement was attributed to enhanced microbial colonization (25), increased digestion in the post-ruminal tract, and subsequently, an increase in the potential degradable fraction and effective degradable content of fiber. These results confirm that the addition of fibrolytic enzyme to normal and slightly lower energy diets improves the digestion of dry matter (DM), organic matter (OM), and NDF.

However, it is important to note that the ingredients used in this trial were high quality, and the digestibility of starch was already relatively high. Therefore, the addition of fibrolytic enzyme may not have further improved the degradability of starch, as it was already 99.17% of DM without enzyme supplementation.

The use of slightly lower energy diets resulted in decreased intake of DM and starch in cows, which is consistent with previous studies that have shown a reduction in dry matter intake (DMI) with a decrease in concentrate amount (6). The substitution of forage with concentrates typically leads to a decrease in intake and digestibility rates. This can be attributed to concentrates causing lesser ruminal fill and having a lower concentration of lignified polysaccharides. The slightly lower energy diet in the current trial tended to reduce total digestibility of DM in the digestive tract (65.93% vs. 67.01%, *p* = 0.07) and OM (68.55% vs. 69.39%, *p =* 0.07). The numerical superiority of the fibrolytic enzyme’s enhancement effect on DM (3.46% vs. 2.58%), NDF (5.52% vs. 4.14%), and pdNDF (7.32% vs. 5.24%) digestibility in the slightly lower energy group compared to the normal energy group was observed. The inclusion of enzymes in the diets of lactating dairy cattle is primarily aimed at improving feed digestion, and therefore, diets with lower digestibility would benefit more from enzyme supplementation (16).

### 4.2 Milk yield and composition

The application of fibrolytic enzymes resulted in increased milk yield, fat-corrected milk (FCM), and energy-corrected milk (ECM) in dairy cows. Previous studies have shown mixed results regarding the effect of fibrolytic enzyme supplementation on milk yield in corn silage-based diets. Some studies reported an increase in milk yield with enzyme addition (8, 9, 25, 26), while others did not observe a milk response (6, 13). These inconsistencies may be attributed to variations in enzyme activity, method of enzyme addition, and differences in the lactation stage of the cows. The increase in milk yield observed in this study can be attributed to improved nutrient utilization in the digestive tract and rumen, leading to an increased net energy gain.

A meta-analysis found that the treatment with exogenous fibrolytic enzymes increased milk yield and milk protein yield, while the effect on feed efficiency (3.5% FCM/DMI) was not significant (4). In the current study, the addition of fibre-degrading enzymes did not affect milk fat concentration, milk protein concentration, milk fat yield and milk protein yield. Changes in milk composition are often associated with alterations in rumen digestion. A study reported that the inclusion of exogenous cellulase in dairy cow diets had no impact on milk fat concentration but increased milk fat yield due to improved fiber digestibility (9). The synthesis of milk fat in bovine mammary glands relies on acetate as the main precursor, which provides carbon and reducing equivalents (NADPH). Some studies have shown that fibrolytic enzymes can influence the ratio of acetate to propionate in the rumen, leading to an increase in milk fat yield (14).

Decreasing the energy levels in the diets resulted in decreased milk yield, FCM, ECM, and milk protein yield. These responses can be attributed to the increase in dietary non-fiber carbohydrates (NFC) concentration as the dietary concentrate level increased (6). Diets with normal energy levels have a high starch content, which promotes the production of propionic acid in the rumen, a precursor for sugar isomerization. This may interact with rumen microbes to produce more microbial proteins or facilitate the flow of nutrients (glucose and amino acids) to the mammary glands, thereby increasing milk and protein yield (27, 28).

The utilization of fibrolytic enzymes in diets has been shown to enhance feed efficiency by reducing the ratios of fat-corrected milk (FCM) to dry matter intake (DMI) and energy-corrected milk (ECM) to DMI. This improvement can be attributed to the increased digestibility of dietary fiber facilitated by the inclusion of fibrolytic enzymes. Cows fed diets with higher fiber digestibility have exhibited greater feed efficiency, characterized by similar DMI but increased milk yield, compared to cows fed diets with lower fiber digestibility (29). These findings provide further confirmation that the supplementation of fibrolytic enzymes in normal and slightly lower energy diets enhances milk production and feed efficiency.

The mean urea nitrogen (MUN) concentration in this study averaged at 16.61 mg/dL, which exceeds the ideal range. MUN serves as a crucial indicator for assessing energy balance and rumen efficiency in dairy cows (30). The elevated MUN levels were attributed to the higher content of rumen-degradable protein in the diet, leading to increased nitrogen excretion. This issue could be addressed by reducing the proportion of rumen-degradable protein in the diet and increasing the inclusion of rumen-undegraded protein. Consistent with previous findings, Jonker (31) reported a positive correlation between MUN and milk yield levels. In the present study, the group receiving the normal lower energy diet exhibited higher levels of urea-N compared to the standard lower energy group (*p* < 0.05). Cows with high milk production often experience a low energy balance, which can be improved through the supplementation of fibrolytic enzymes.

### 4.3 Plasma nutrients

In the study, plasma free amino acid (FAA) concentrations were found to be significantly higher in the normal energy group compared to the slightly lower energy group (10.14 vs. 7.47 uM, *p* < 0.001). The synthesis of milk protein in the mammary gland of dairy cows is a complex metabolic process, and amino acids are transferred to the mammary gland mainly through plasma (32). Free amino acids act as signaling molecules that promote milk protein synthesis and serve as substrates for mammary uptake from the blood for milk protein synthesis. Therefore, the concentration of FAA in plasma regulates the milk protein concentration and yield in dairy cows (33). The study found that plasma FAA concentration was significantly lower in cows fed the slightly lower energy diet compared to cows fed the normal energy diet (*p* < 0.001), and milk protein yield was also lower in the slightly lower energy group compared to the normal energy group (*p* = 0.037).

Plasma β-hydroxybutyrate (BHBA) concentrations varied with substrate energy levels following the addition of fibrolytic enzymes to the diets of dairy cows (enzyme * energy interaction, *p* < 0.001). The normal energy group had significantly lower plasma BHBA concentrations than the slightly lower energy group (0.1 vs. 0.13 mM, *p* < 0.001). There was a trend toward lower plasma glucose (GLU) levels in the group receiving fibrolytic enzymes (4.01 vs. 4.09 mM, *p* = 0.099). Changes in plasma GLU and BHBA concentrations indicate changes in the energy status of cows. BHBA, derived from microbial fermentation in the rumen, plays a crucial role in *de novo* lipogenesis within the mammary gland (34). The addition of fibrolytic enzymes to the normal energy group resulted in a decrease in plasma BHBA concentration (*p* < 0.05), consistent with other studies (20, 35), where improving cow energy status through the use of fibrolytic cellulases led to reduced plasma BHBA concentrations, indicating decreased mobilization of fat from adipose tissue in lactating dairy cows. However, the addition of fibrolytic enzymes in the slightly lower energy group resulted in an increase in plasma BHBA concentrations. Combined with trends in plasma glucose concentrations, this may indicate that cows fed a slightly lower energy diet with fibrolytic enzymes mobilized muscle metabolism alongside body fat for energy in order to maintain high milk production. Additionally, β-hydroxybutyric acid has been reported to up-regulate the expression of milk protein synthesis genes in mammary epithelial tissues (36), which may partly explain the enhancement of milk protein yield in the slightly lower energy group with the addition of fibrolytic enzymes (1.48 vs. 1.45 kg/d). The addition of fibrolytic enzymes to diets at different energy levels resulted in different plasma BHBA concentrations and different milk component yields, suggesting that different dietary energies lead to different pathways by which cellulase enhances cow performance.

## 5 Conclusion

Adding fibrolytic enzymes (mixture of xylanase and cellulase) to the diets of high-producing dairy cows can improve nutrient digestibility, milk yield and feed efficiency. Enzyme application to the slightly lower energy diet resulted in as much milk production as that from cows fed the untreated normal diet.

## Data availability statement

The original contributions presented in the study are included in the article/Supplementary material, further inquiries can be directed to the corresponding author.

## Ethics statement

The use of the animals and the experimental procedures were approved by the Animal Care Committee of Institute of Animal Sciences, Chinese Academy of Agricultural Sciences (Beijing, China) in accordance with the guidelines for animal experimental welfare and ethical inspection in China (No: IAS 2019–28). The studies were conducted in accordance with the local legislation and institutional requirements. Written informed consent was obtained from the owners for the participation of their animals in this study.

## Author contributions

JY: Data curation, Investigation, Project administration, Writing – original draft, Writing – review & editing. SZ: Conceptualization, Data curation, Formal analysis, Funding acquisition, Investigation, Supervision, Writing – review & editing. BL: Conceptualization, Data curation, Formal analysis, Supervision, Writing – review & editing.
